# Organizational Implementation Capacity of Vocational Rehabilitation During Secondary Transition for Individuals with Intellectual Disabilities: A Preliminary Study in Al-Kharj, Saudi Arabia

**DOI:** 10.3390/jintelligence14090230

**Published:** 2026-09-21

**Authors:** Hussain A. Almalky, Arwa M. Alwadei

**Affiliations:** 1 Department of Special Education, College of Education in Al-Kharj, Prince Sattam bin Abdulaziz University, Al-Kharj 11942, Saudi Arabia; 2 Ministry of Education, Riyadh 11148, Saudi Arabia; arwa.m.w@hotmail.com

**Keywords:** vocational rehabilitation, intellectual disabilities, implementation capacity, adaptive functioning, secondary transition, school-to-work transition, Al-Kharj, Saudi Arabia

## Abstract

Vocational rehabilitation (VR) plays an important role in supporting successful school-to-work transitions for individuals with intellectual disabilities (IDs). However, the organizational conditions that enable effective implementation of VR practices remain insufficiently understood. Because participation outcomes among individuals with ID are influenced by interactions between intellectual functioning, adaptive abilities, and environmental supports, examining organizational implementation capacity provides an important systems-level perspective. This study examined professionals’ perceptions of organizational implementation capacity for VR during secondary transition in Saudi Arabia and explored differences according to institutional and professional characteristics. A cross-sectional survey was conducted among 89 professionals working in government secondary school programs and specialized education centers in Al-Kharj, Saudi Arabia. Participants completed the Vocational Rehabilitation Implementation Capacity Measure (VRICM), a context-specific, theoretically informed measure developed for this study. The VRICM examines four theoretically derived areas: Support and Related Services, Vocational Preparation Services, Collaborative Planning, and Workplace-Based Training. We conducted descriptive statistics and nonparametric group comparisons. Overall perceived organizational implementation capacity was moderate (M = 3.25, SD = 0.95). Support and Related Services and Vocational Preparation Services received higher ratings than Collaborative Planning and Workplace-Based Training. Differences were observed according to institutional sector and professional experience. Findings provide preliminary evidence regarding organizational factors supporting VR implementation within a Saudi Arabian secondary-city context. Further psychometric evaluation of the VRICM, including structural validity assessment, is required before broader application.

## 1. Introduction

Employment represents an important aspect of social participation, independence, and quality of life for individuals with intellectual disabilities (IDs). Individuals with ID often experience challenges in employment participation because of the complex interaction between intellectual functioning, adaptive behavior, educational opportunities, workplace accessibility, and availability of appropriate support systems (Nevala et al., 2019; Voermans et al., 2021; World Health Organization, 2011). Although individual characteristics related to intellectual functioning and adaptive abilities are important, contemporary disability perspectives emphasize that participation outcomes are also shaped by environmental supports, service systems, and organizational capacity.

Individuals with ID continue to experience lower employment participation rates compared with the general population despite increasing recognition of their rights to meaningful participation in education, employment, and community life (Nevala et al., 2019). Therefore, understanding employment outcomes requires attention not only to individual characteristics but also to the systems responsible for providing transition and vocational supports.

The transition from school to employment represents a critical developmental period for individuals with ID. Successful transition requires coordinated planning among students, families, educational institutions, rehabilitation professionals, employers, and community agencies (Carter et al., 2012; Test et al., 2009; Wehman et al., 2020). Vocational rehabilitation (VR) plays an important role in facilitating this transition through services such as vocational assessment, career planning, workplace preparation, employment training, and ongoing employment support (Kohler et al., 2016; Test et al., 2009).

However, the availability of vocational rehabilitation services alone does not guarantee successful transition outcomes. The extent to which organizations can implement coordinated, evidence-informed vocational rehabilitation practices may influence the opportunities available to individuals with ID.

Research indicates that effective transition programs depend on multiple interacting components, including individualized planning, student development, family involvement, interagency collaboration, and program structures (Kohler et al., 2016). Despite evidence supporting these practices, implementation remains inconsistent across educational and rehabilitation settings (Lambert et al., 2023; Schall et al., 2024).

### 1.1. Implementation Capacity in Vocational Rehabilitation

Implementation science provides a framework for understanding why evidence-informed practices are successfully adopted and sustained in some settings but not others (Nilsen & Birken, 2020). Rather than focusing only on whether an intervention or service is effective, implementation science examines the organizational and contextual conditions that influence whether effective practices can be delivered consistently in real-world settings.

Organizational implementation capacity refers to the extent to which organizations possess the structures, resources, leadership support, communication processes, and collaborative systems necessary to implement and sustain evidence-informed practices (Castruita Rios et al., 2023; Damschroder et al., 2022; Nilsen & Birken, 2020).

The Consolidated Framework for Implementation Research (CFIR) provides a comprehensive framework for examining factors that influence implementation across multiple levels, including organizational characteristics, available resources, internal processes, external partnerships, and broader contextual influences (Damschroder et al., 2022). Within vocational rehabilitation settings, CFIR provides a useful perspective for understanding organizational conditions that may facilitate or constrain implementation.

Although CFIR explains factors influencing implementation, it does not define the specific content of effective secondary transition programming. Therefore, this study also draws upon the Kohler Taxonomy for Transition Programming, which identifies essential components of high-quality transition services, including student-focused planning, student development, family involvement, interagency collaboration, and program structures (Kohler et al., 2016).

In this study, CFIR and the Kohler Taxonomy were used as complementary theoretical foundations. The Kohler Taxonomy informed the identification of transition-related vocational rehabilitation practices examined within the VRICM, whereas CFIR informed interpretation of organizational conditions that may influence implementation capacity. These frameworks were not intended to represent a newly integrated theoretical model but were used to provide a conceptual basis for examining perceived organizational implementation capacity.

### 1.2. Vocational Rehabilitation Context in Saudi Arabia and the Gulf Region

Within Saudi Arabia, disability inclusion and employment participation have received increasing policy attention through national initiatives aimed at improving education, rehabilitation, and workforce participation opportunities for individuals with disabilities. The Rights of Persons with Disabilities Law established a national legislative framework supporting the rights, services, and inclusion of persons with disabilities, including access to rehabilitation, training, and employment opportunities (Bureau of Experts at the Council of Ministers, 2023). In addition, the Ministry of Human Resources and Social Development has introduced initiatives supporting vocational preparation, workplace inclusion, and labor market participation among individuals with disabilities (Ministry of Human Resources and Social Development, n.d.). These developments reflect a broader commitment toward improving participation outcomes; however, translating policy objectives into effective vocational outcomes requires coordinated service systems, appropriate rehabilitation pathways, and organizational structures that support consistent implementation.

Recent Saudi research has highlighted persistent challenges affecting employment participation and vocational development among individuals with disabilities. Evidence suggests that barriers remain related to employment accessibility, workplace opportunities, vocational preparation, and coordination between relevant stakeholders (Abed et al., 2024; Alasmari & Almalky, 2026). Within the context of intellectual disability, research has identified additional challenges related to the relevance of vocational curricula, career readiness, and the availability of structured transition services that facilitate movement from school-based programs to adult employment settings (Aldossari et al., 2024; Almalky & Alwadei, 2024).

Although these studies provide important insight into disability employment and vocational service challenges in Saudi Arabia, the existing literature has largely focused on describing barriers, service availability, or employment-related outcomes. Less attention has been directed toward understanding the organizational conditions that influence how vocational rehabilitation practices are implemented within educational and rehabilitation settings. Successful transition outcomes require more than the availability of individual services; they depend on effective collaboration among schools, rehabilitation professionals, families, employers, and community organizations, as well as organizational readiness to deliver coordinated and sustainable services.

Similar challenges have been reported internationally, where transition outcomes for individuals with intellectual disabilities are strongly associated with the quality of transition planning, stakeholder collaboration, workplace-based learning opportunities, and employment supports (Kohler et al., 2016; Test et al., 2009). International evidence further indicates that employment participation is influenced not only by individual abilities but also by environmental and organizational factors that shape opportunities for participation and inclusion (Nevala et al., 2019). Within the Gulf region, disability services have increasingly emphasized educational inclusion and vocational participation; however, empirical research examining the organizational implementation processes underlying vocational rehabilitation remains limited. This limitation highlights the importance of investigating how rehabilitation practices are organized, supported, and delivered within specific cultural and service contexts.

From an implementation science perspective, evidence-informed practices are more likely to be successfully adopted and sustained when organizations demonstrate adequate readiness, leadership support, resource availability, communication processes, and external partnerships (Damschroder et al., 2022; Nilsen & Birken, 2020). Therefore, examining organizational implementation capacity provides an important approach for understanding the factors that may facilitate or constrain vocational rehabilitation delivery during secondary transition. This perspective is particularly relevant in emerging rehabilitation systems, such as those within Saudi Arabia, where understanding organizational conditions may contribute to improving transition services and participation outcomes for individuals with intellectual disabilities.

### 1.3. Theoretical Framework and Study Rationale

Transition from secondary education to adult life represents a complex process that requires coordinated planning and collaboration among multiple stakeholders. For individuals with intellectual disabilities, successful transition outcomes are influenced by interactions between individual characteristics, adaptive functioning, available supports, and environmental opportunities. Consequently, vocational rehabilitation should be understood not only as a set of individual interventions but also as a coordinated system involving educational, rehabilitation, and employment-related structures.

The Kohler Taxonomy for Transition Programming provides a comprehensive framework for understanding effective transition practices by emphasizing student-focused planning, student development, family involvement, interagency collaboration, and program structures (Kohler et al., 2016). This framework highlights the importance of coordinated transition services that prepare individuals with disabilities for meaningful participation beyond secondary education. However, the presence of recommended transition practices alone does not guarantee successful implementation, as organizational and contextual factors may influence whether these practices are consistently delivered.

Implementation science provides additional insight into these organizational processes. The Consolidated Framework for Implementation Research (CFIR) identifies multiple domains that influence implementation success, including intervention characteristics, inner organizational context, outer contextual influences, characteristics of individuals involved, and implementation processes (Damschroder et al., 2022). Applying an implementation perspective to vocational rehabilitation allows researchers to examine not only which practices are provided but also the organizational conditions that support or limit their delivery.

In the current study, the Kohler Taxonomy and CFIR were used as complementary conceptual foundations. The Kohler Taxonomy informed the identification of key vocational rehabilitation components relevant to secondary transition, whereas CFIR guided interpretation of organizational and contextual factors influencing implementation capacity. These frameworks were not examined as an integrated theoretical model; rather, they provided a structured basis for exploring professionals’ perceptions of organizational capacity to implement vocational rehabilitation practices.

Despite growing attention to disability inclusion and employment participation, limited research has examined organizational implementation capacity for vocational rehabilitation during secondary transition, particularly within the Saudi Arabian context. Understanding professionals’ perceptions of implementation capacity may provide valuable insight into current strengths and areas requiring further development within educational and rehabilitation systems. Therefore, the present study aimed to examine professionals’ perceived organizational implementation capacity for vocational rehabilitation during secondary transition for individuals with intellectual disabilities in Al-Kharj, Saudi Arabia.

## 2. Materials and Methods

### 2.1. Study Design and Participants

This study employed a cross-sectional survey design to examine professionals’ perceptions of organizational implementation capacity for vocational rehabilitation (VR) during secondary transition for individuals with intellectual disabilities (IDs). The study was conducted in Al-Kharj, Saudi Arabia, a secondary city with educational and rehabilitation services supporting students with ID. The study focused on professionals working in government secondary school programs and specialized education centers who were directly involved in transition planning, vocational preparation, rehabilitation services, special education, or related support activities.

Participants were eligible if they had professional experience supporting students with ID during secondary transition or vocational rehabilitation processes. The purpose of the study was to examine perceived organizational implementation capacity within a specific service context rather than to evaluate the effectiveness of vocational rehabilitation interventions or employment outcomes. Accordingly, findings represent professionals’ perceptions of organizational readiness and implementation conditions within the studied settings.

### 2.2. Development of the Vocational Rehabilitation Implementation Capacity Measure (VRICM)

The Vocational Rehabilitation Implementation Capacity Measure (VRICM) was developed specifically for this study to examine professionals’ perceptions of organizational capacity to implement vocational rehabilitation practices during secondary transition for individuals with ID. The measure was developed as a context-specific, theoretically informed instrument based on established transition programming literature and implementation science principles.

The conceptual development of the VRICM was guided by two complementary frameworks: the Kohler Taxonomy for Transition Programming and the Consolidated Framework for Implementation Research (CFIR). The Kohler Taxonomy was used to identify key components of effective secondary transition programming, including student-focused planning, student development, family involvement, interagency collaboration, and program structures. In contrast, CFIR was used to provide a broader implementation science perspective by considering organizational and contextual factors that may facilitate or constrain the adoption and delivery of evidence-informed practices.

Therefore, the two frameworks served complementary purposes within this study. The Kohler Taxonomy informed the identification of vocational rehabilitation-related practices examined through the VRICM, whereas CFIR guided interpretation of organizational implementation conditions. These frameworks were not tested as an integrated theoretical model but were used to provide a conceptual foundation for examining perceived implementation capacity.

### 2.3. VRICM Structure and Content

The VRICM consists of 17 items organized into four theoretically derived areas: Support and Related Services, Vocational Preparation Services, Collaborative Planning, and Workplace-Based Training. These areas reflect key components of vocational rehabilitation implementation during secondary transition and were derived from relevant transition and implementation literature.

Participants rated each item using a Likert-type response scale, with higher scores indicating higher perceived organizational implementation capacity. The total VRICM score was calculated to represent overall perceived implementation capacity, while area-specific scores were examined to explore variation across different components of vocational rehabilitation implementation.

Although the VRICM was developed using established theoretical frameworks and demonstrated preliminary reliability evidence in the current sample, it should not be considered a fully validated psychometric instrument. Internal consistency alone does not establish construct validity; therefore, future research should examine the structural validity of the VRICM using larger and more diverse samples through appropriate psychometric procedures, including exploratory and confirmatory factor analyses.

### 2.4. Data Collection Procedure

Data collection was conducted following institutional ethical approval procedures. Participants were informed about the purpose of the study, confidentiality procedures, voluntary participation, and their right to withdraw at any time without consequences. After providing informed consent, participants completed the VRICM based on their professional experiences within their respective educational or rehabilitation settings.

Because the VRICM assesses professional perceptions, the scores represent perceived organizational implementation capacity rather than objective measures of organizational performance. This approach was selected because professionals directly involved in transition services provide valuable insight into organizational processes, collaboration structures, and implementation challenges.

### 2.5. Statistical Analysis

Descriptive statistics were calculated to summarize participant characteristics and VRICM responses. Means and standard deviations were used to describe overall perceived implementation capacity and scores across the four theoretically derived areas.

The distribution of the data was examined, and nonparametric statistical procedures were applied where assumptions required for parametric testing were not met. Differences between two groups were examined using the Mann–Whitney U test, whereas comparisons involving more than two groups were conducted using the Kruskal–Wallis test. Where statistically significant differences were identified, post hoc comparisons were performed using Dunn’s test with Holm adjustment to control for multiple comparisons. Because Mann–Whitney U analyses were conducted as exploratory two-group comparisons based on predefined participant characteristics, these comparisons were interpreted cautiously, and emphasis was placed on effect sizes and consistency of findings rather than isolated statistical significance.

Statistical findings were interpreted as associations between participant characteristics and perceived organizational implementation capacity rather than causal relationships. In addition, subgroup comparisons were interpreted cautiously because unequal subgroup sizes may influence statistical power and the stability of group comparisons.

### 2.6. Ethical Considerations

Ethical approval was obtained before data collection. Participation was voluntary, and all participants provided informed consent before completing the survey. Data were collected anonymously and handled according to institutional research ethics requirements.

## 3. Results

### 3.1. Participant Characteristics

A total of 89 professionals participated in the study. Participants were recruited from government secondary school programs and specialized education centers providing services for individuals with intellectual disabilities in Al-Kharj, Saudi Arabia. Participant characteristics, including gender, educational qualification, professional experience, and institutional sector, are presented in Table 1.

### 3.2. Descriptive Statistics of the VRICM

The overall VRICM score indicated a moderate level of perceived organizational implementation capacity for vocational rehabilitation during secondary transition (M = 3.25, SD = 0.95). Across the four theoretically derived areas of the VRICM, Support and Related Services demonstrated the highest perceived implementation capacity (M = 3.64, SD = 1.05), followed by Vocational Preparation Services (M = 3.40, SD = 0.88). Collaborative Planning (M = 3.11, SD = 0.84) and Workplace-Based Training (M = 2.85, SD = 1.02) received comparatively lower ratings. Item-level descriptive statistics for the VRICM areas are presented in Table 2.

### 3.3. Differences in VRICM Scores According to Participant Characteristics

Nonparametric analyses were conducted to examine differences in perceived organizational implementation capacity according to institutional sector, gender, educational qualification, and professional experience.

#### 3.3.1. Institutional Sector, Gender, and Educational Qualification

Institutional sector was significantly associated with perceived organizational implementation capacity. Professionals working in specialized education centers reported significantly higher overall VRICM scores compared with professionals working in government secondary school programs (U = 454.00, Z = −4.06, *p* < .001, r = 0.43).

Significant differences between institutional sectors were also observed for Support and Related Services (U = 306.50, Z = −5.34, *p* < .001, r = 0.57), Collaborative Planning (U = 578.50, Z = −3.03, *p* = .002, r = 0.32), and Vocational Preparation Services (U = 558.00, Z = −3.20, *p* = .001, r = 0.34). No significant difference was identified for Workplace-Based Training (U = 778.00, Z = −1.33, *p* = .183, r = 0.14).

Gender differences were generally not observed across VRICM areas. A significant difference was identified only for Workplace-Based Training, where male professionals reported higher perceived implementation capacity than female professionals (U = 496.50, Z = −2.47, *p* = .013, r = 0.26). No significant gender differences were observed for the overall VRICM score or the remaining areas.

Educational qualification was not significantly associated with perceived organizational implementation capacity. Professionals with bachelor’s degrees and graduate qualifications reported comparable overall VRICM scores (U = 413.00, Z = −0.94, *p* = .346, r = 0.10).

Detailed results are presented in Table 3.

#### 3.3.2. Professional Experience

Kruskal–Wallis analyses revealed statistically significant differences in perceived organizational implementation capacity according to professional experience across all VRICM areas and the overall VRICM score. Significant differences were observed for the overall VRICM score (χ^2^(2) = 20.09, *p* < .001, ε^2^ = 0.23), Support and Related Services (χ^2^(2) = 17.52, *p* < .001, ε^2^ = 0.20), Collaborative Planning (χ^2^(2) = 21.86, *p* < .001, ε^2^ = 0.25), Vocational Preparation Services (χ^2^(2) = 9.38, *p* = .009, ε^2^ = 0.11), and Workplace-Based Training (χ^2^(2) = 17.04, *p* < .001, ε^2^ = 0.19).

Post hoc Dunn tests with Holm adjustment indicated that professionals with ≤5 years of experience reported higher perceived implementation capacity than those with 6–10 years of experience across the overall VRICM score and multiple areas. Professionals with ≥11 years of experience generally demonstrated intermediate levels of perceived implementation capacity, with significant differences observed for selected comparisons.

Overall, these findings indicate a nonlinear association between professional experience and perceived organizational implementation capacity, rather than a consistent increase or decrease across experience categories.

Detailed results are presented in Table 4 and Table 5.

## 4. Discussion

The present study examined professionals’ perceptions of organizational implementation capacity for vocational rehabilitation (VR) during secondary transition for individuals with intellectual disabilities (IDs) in Al-Kharj, Saudi Arabia. Overall, participants reported a moderate level of perceived implementation capacity, suggesting that vocational rehabilitation practices are present within the studied settings but may require further organizational strengthening to support more consistent implementation.

The findings contribute to the growing literature on intellectual disability and participation by highlighting that successful transition outcomes are not determined solely by individual characteristics, including intellectual functioning and adaptive abilities, but are also shaped by the availability and quality of environmental supports. From an ecological perspective of disability, opportunities for employment participation depend on interactions between individuals and the systems surrounding them, including educational institutions, rehabilitation providers, employers, and community networks (World Health Organization, 2011; Nevala et al., 2019).

### 4.1. Interpretation of VR Implementation Capacity Findings

Among the four theoretically derived areas examined by the VRICM, Support and Related Services and Vocational Preparation Services received relatively higher ratings, whereas Collaborative Planning and Workplace-Based Training received comparatively lower ratings.

The relatively stronger perceptions of support services may indicate that professionals recognize the importance of providing foundational assistance during transition planning. However, lower perceptions related to collaborative planning and workplace-based training highlight areas where organizational coordination and external partnerships may require additional attention. “This is consistent with evidence that employer practices, workplace accessibility, and reasonable adjustments influence employment opportunities for individuals with disabilities (Olsen, 2024).”

Previous research has demonstrated that effective transition programming requires coordinated involvement among schools, families, rehabilitation providers, and employers (Kohler et al., 2016; Test et al., 2009). Therefore, challenges in collaboration and workplace-based experiences may represent broader implementation barriers rather than limitations associated with individual professional commitment.

### 4.2. Organizational Implementation Capacity and Theoretical Interpretation

The findings support the importance of examining vocational rehabilitation implementation through an organizational lens. Although transition frameworks identify essential service components, implementation science emphasizes that the presence of recommended practices does not necessarily guarantee consistent delivery. The current study used the Kohler Taxonomy and CFIR as complementary conceptual foundations. The Kohler Taxonomy helped identify relevant transition-related vocational rehabilitation practices, whereas CFIR provided a framework for interpreting organizational and contextual conditions that may support or constrain implementation (Damschroder et al., 2022; Kohler et al., 2016). Importantly, this study did not test CFIR constructs statistically or establish a new integrated theoretical model. Instead, these frameworks were used to guide interpretation of professionals’ perceptions regarding organizational implementation capacity.

### 4.3. Differences According to Institutional and Professional Characteristics

Differences in perceived organizational implementation capacity were observed according to institutional sector and professional experience.

Institutional differences may reflect variation in organizational resources, service structures, leadership support, communication pathways, and opportunities for collaboration. These findings align with implementation science research indicating that organizational context plays an important role in whether evidence-informed practices are adopted and sustained (Damschroder et al., 2022; Nilsen & Birken, 2020).

A nonlinear pattern was observed across professional experience groups. Although differences were identified, these findings should be interpreted cautiously. The cross-sectional design does not allow conclusions regarding causal relationships, and unequal subgroup sizes may influence statistical power. Future research using larger and more balanced samples is needed to further examine how professional characteristics relate to implementation capacity.

### 4.4. Implications for Vocational Rehabilitation Practice

The findings have several implications for vocational rehabilitation services supporting individuals with ID. First, strengthening organizational capacity may require greater emphasis on coordinated transition planning involving educational institutions, families, rehabilitation professionals, and employers. Second, workplace-based training opportunities may require additional organizational investment. Evidence suggests that employment participation among individuals with disabilities is improved when services include meaningful workplace experiences, employer engagement, individualized support, and evidence-based supported employment approaches (Bonaccio et al., 2020; Drake & Bond, 2023; Nevala et al., 2019; Sundermann et al., 2023). Third, implementation efforts should consider contextual factors rather than focusing exclusively on individual-level skills. Building sustainable vocational rehabilitation systems requires attention to organizational readiness, available resources, communication processes, and external partnerships.

### 4.5. Limitations and Future Research

Several limitations should be considered when interpreting these findings. First, the study was conducted within a specific Saudi Arabian secondary-city context. Therefore, findings may not be directly generalizable to other regions or countries with different educational, rehabilitation, and employment systems. Second, the study relied on professionals’ self-reported perceptions. Although these perspectives provide valuable insight into organizational conditions, they do not represent objective measurements of organizational performance or employment outcomes. Third, the VRICM should be considered a context-specific, theoretically informed measure rather than a fully validated instrument. Although the study provides preliminary evidence regarding perceived implementation capacity, future research should examine the measure’s psychometric properties, including structural validity through exploratory and confirmatory factor analyses using larger and more diverse samples. Future studies should also examine implementation capacity across multiple geographic regions and investigate how organizational conditions relate to actual transition outcomes, including employment participation and long-term vocational success.

### 4.6. Contribution of the Study

This study contributes preliminary evidence regarding vocational rehabilitation implementation by emphasizing the importance of considering organizational capacity alongside individual-level characteristics when examining participation opportunities for individuals with intellectual disabilities. By examining professionals’ perceptions within a Saudi Arabian context, the study provides initial insight into organizational factors that may influence the implementation of transition-related vocational rehabilitation practices.

## 5. Conclusions

The present study examined professionals’ perceptions of organizational implementation capacity for vocational rehabilitation during secondary transition for individuals with intellectual disabilities in Al-Kharj, Saudi Arabia. Findings indicated a moderate level of perceived implementation capacity, with variation across different vocational rehabilitation implementation areas and selected professional and institutional characteristics. The study highlights the importance of examining vocational rehabilitation implementation beyond individual-level factors by considering the organizational and contextual conditions that shape opportunities for participation. In particular, effective transition support for individuals with intellectual disabilities requires coordinated services, collaborative planning, workplace-based experiences, and organizational structures that facilitate consistent implementation.

The findings provide preliminary insight into vocational rehabilitation implementation capacity within a Saudi Arabian secondary-city context. However, interpretations should consider the study’s contextual limitations, cross-sectional design, and reliance on professional perceptions and the preliminary psychometric status of the VRICM. Future research should examine the VRICM using larger and more diverse samples, including formal evaluation of structural validity through exploratory and confirmatory factor analyses. Additional research should also investigate how organizational implementation capacity relates to objective transition outcomes, including employment participation and long-term vocational success. Overall, this study contributes to understanding the organizational conditions that may support vocational rehabilitation implementation for individuals with intellectual disabilities and provides a foundation for future research examining how service systems can better promote participation and inclusion.

## Figures and Tables

**Table 1:** Demographic and Professional Characteristics of Participants (*n* = 89).

Variable	Category	Frequency (*n*)	Percentage (%)
Gender	Male	23	25.8
	Female	66	74.2
Educational qualification	Bachelor’s degree	76	85.4
	Graduate studies	13	14.6
Professional experience	≤5 years	35	39.3
	6–10 years	35	39.3
	≥11 years	19	21.4
Institutional sector	Government secondary school program	55	61.8
	Specialized education center	34	38.2

**Table 2:** Descriptive Statistics for the VRICM Theoretically Derived Areas and Items.

VRICM Area/Item	M	SD
Support and Related Services	3.64	1.05
Psychological support services are delivered by qualified psychologists	3.96	0.98
Ancillary services, including physical and speech therapy, are available	3.58	1.04
Social support services are delivered by professional social workers	3.56	1.12
Assistive technologies are accessible to enhance training processes	3.45	1.25
Vocational Preparation Services	3.40	0.88
Individuals with ID receive training tailored to their abilities	3.69	1.07
The institution offers vocational guidance and counseling services	3.45	1.12
A range of occupational training opportunities is provided	3.24	1.03
Vocational interest and aptitude assessments are systematically administered	3.22	1.17
Collaborative Planning	3.11	0.84
The rehabilitation process undergoes regular evaluation	3.52	1.08
Objectives align with the interests and preferences of individuals	3.49	1.17
Families are actively involved throughout the rehabilitation process	3.46	1.08
Individuals with ID participate in the preparation of their vocational portfolios	2.79	1.07
Individuals with ID are involved in planning sessions for employment strategies	2.65	1.09
Workplace-Based Training	2.85	1.02
Visits to real workplace settings are organized for exploration and training	3.01	1.26
Vocational training is designed to address current labor market demands	2.91	1.03
Employers are engaged in the training of individuals with ID	2.82	1.23
Employers are encouraged to provide training contracts that facilitate subsequent employment	2.64	1.13

Note. Higher scores indicate higher perceived organizational implementation capacity. The four VRICM areas represent theoretically derived areas informed by transition programming and implementation science frameworks.

**Table 3:** Differences in VRICM Scores According to Institutional Sector, Gender, and Educational Qualification.

Comparison	VRICM Area	U	Z	*p*	Effect Size (r)
Institutional Sector	Support and Related Services	306.50	−5.34	<.001 *	0.57
	Collaborative Planning	578.50	−3.03	.002 *	0.32
	Vocational Preparation Services	558.00	−3.20	.001 *	0.34
	Workplace-Based Training	778.00	−1.33	.183	0.14
	Overall VRICM Score	454.00	−4.06	<.001 *	0.43
Gender	Support and Related Services	728.00	−0.29	.770	0.03
	Collaborative Planning	685.50	−0.69	.489	0.07
	Vocational Preparation Services	719.50	−0.37	.710	0.04
	Workplace-Based Training	496.50	−2.47	.013 *	0.26
	Overall VRICM Score	732.50	−0.25	.804	0.03
Educational Qualification	Overall VRICM Score	413.00	−0.94	.346	0.10

Note. Mann–Whitney U tests were used for comparisons between two groups. * *p* ≤ .05. Effect size (r) represents rank-biserial correlation.

**Table 4:** Differences in VRICM Scores According to Professional Experience.

VRICM Area	≤5 Years Mean Rank	6–10 Years Mean Rank	≥11 Years Mean Rank	χ^2^(df = 2)	*p*	ε^2^
Support and Related Services	58.71	33.44	41.03	17.52	<.001 *	0.20
Collaborative Planning	58.79	30.16	46.95	21.86	<.001 *	0.25
Vocational Preparation Services	53.53	35.00	47.71	9.38	.009 *	0.11
Workplace-Based Training	52.23	31.21	57.08	17.04	<.001 *	0.19
Overall VRICM Score	58.37	30.77	46.58	20.09	<.001 *	0.23

Note. Kruskal–Wallis tests were used to compare professional experience groups. * *p* ≤ .05.

**Table 5:** Post Hoc Pairwise Comparisons of VRICM Scores by Professional Experience Groups.

VRICM Area	≤5 Years vs. 6–10 Years	≤5 Years vs. ≥11 Years	6–10 Years vs. ≥11 Years
Support and Related Services	<0.001 *	0.016 *	0.300
Collaborative Planning	<0.001 *	0.106	0.022 *
Vocational Preparation Services	0.008 *	0.427	0.082
Workplace-Based Training	0.001 *	0.508	0.001 *
Overall VRICM Score	<0.001 *	0.109	0.032 *

Note. *p* values were adjusted using the Holm correction. * *p* ≤ .05.

## Data Availability

The data that support the findings of this study are available from the corresponding author upon reasonable request.

## Abbreviations

The following abbreviations are used in this manuscript:

VR	Vocational Rehabilitation
ID	Intellectual Disability
CFIR	The Consolidated Framework for Implementation Research
VRICM	Vocational Rehabilitation Implementation Capacity Measure
